# Supplementary material for: Large-scale groundwater flow and sedimentary diagenesis in continental shelves influence marine chemical budgets
Source: Nat Commun. 2024 Feb 7;15:1143. doi: 10.1038/s41467-024-44919-7 (PMC10850067; doi:10.1038/s41467-024-44919-7)
Supplement: Supplementary file 1 — Supplementary Information [file 41467_2024_44919_MOESM1_ESM.pdf]

Supplementary Information

Large-scale groundwater flow and sedimentary diagenesis in continental shelves influence marine chemical budgets

Alicia M. Wilson[1*], Andrew Osborne[1,2], and Scott M. White[1]

[1]School of the Earth, Ocean and Environment
University of South Carolina, Columbia SC 29208, USA
[2]Now at: INTERA, 3 Sugar Creek Center Blvd., Suite 675, Sugar Land, TX 77478, USA
[*]awilson@seoe.sc.edu

Supplementary Table 1

Supplementary Table 1. Calculations showing adjustment of original groundwater compositions to calculate net fluxes.

	Ca^{2+}	K^{+}	Mg^{2+}	Na^{+}	Cl^{-}
			Type 3		
1	2260.8	211.6	549.7	2097.6	5132.8
2	241.3	22.6	58.7	223.9	547.9
3	20.6	10.3	106.6	469.7	547.9
4	220.7	12.3	-48.0	-245.8	0
5	110.4	12.3	-24.0	-245.8	0
			MOR		
6	76.3	37.3	1.3	418	532.3
7	78.5	38.39314	1.3	430.3	547.9
8	20.6	10.3	106.6	469.7	547.9
9	57.9	28.1	-105.2	-39.4	0
10	29.0	28.1	-52.6	-39.4	0

1 — Original Type 3 brine fluid (meq/L)
2 — Adjust to Seawater Cl^{-}
3 — Seawater
4 — Net Chemical Contribution (meq/L)
5 — Net Chemical Contribution (mmol/L)
6 — MOR composition (meq/L)
7 — Adjust to Seawater Cl^{-}
8 — Seawater
9 — Net MOR Contribution (meq/L)
10 — Net MOR Contribution (mmol/L)

Supplementary Table 2

Supplementary Table 3. Calculated[a] and observed[b] compositions of discharge from low-temperature hydrothermal alteration, normalized relative to initial seawater composition (mmol/L)

	Ca^{2+}	K^{+}	Mg^{2+}	Na^{+}
Calculated				
Case 1	1.40	-0.137	-0.070	-0.262
Case 2	1.28	-0.160	-0.079	0.062
Observed				
Dorado outcrop	-0.04	-0.4	-0.2	0
Baby Bare	45.0	-3.29	-51.6	11

[a]Global average composition calculated based on Equation 4. Case 1 uses the clastic-derived archetype for C_S. Case 2 uses the $CaCl_2$ brine archetype for C_S.
[b]Adjusted compositions from samples collected at individual field sites.

Supplementary Figure 1

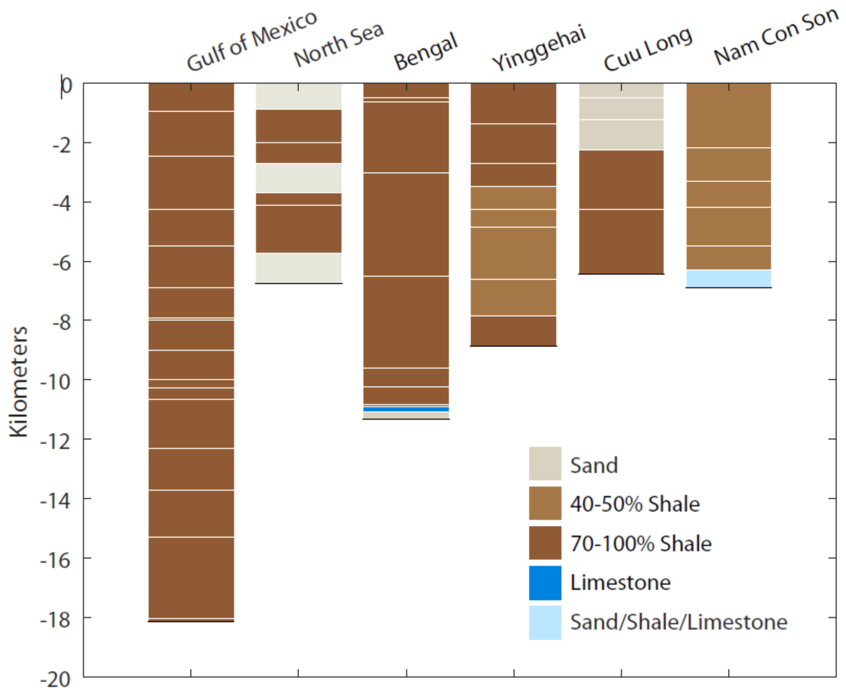

Supplementary Figure 1. Sediment columns from the six basins representative of compaction-driven flow. Stratigraphy compiled from the sources listed in Table 2. Source data are provided as a Source Data file.

Supplementary Figure 2

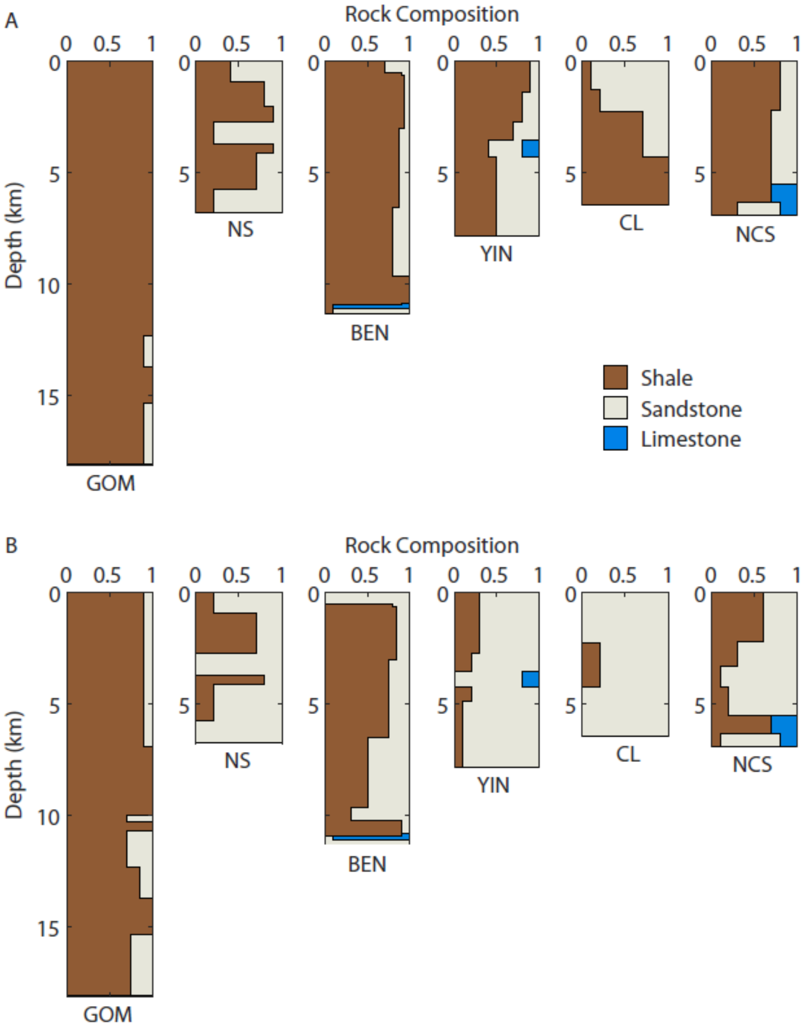

Supplementary Figure 2. Shale, sandstone, and limestone content of the six sedimentary basins representative of compaction-driven flow. (a) Maximum shale content. (b) Minimum shale content. Stratigraphy compiled from the sources listed in Table 2. Source data are provided as a Source Data file.

Supplementary Figure 3

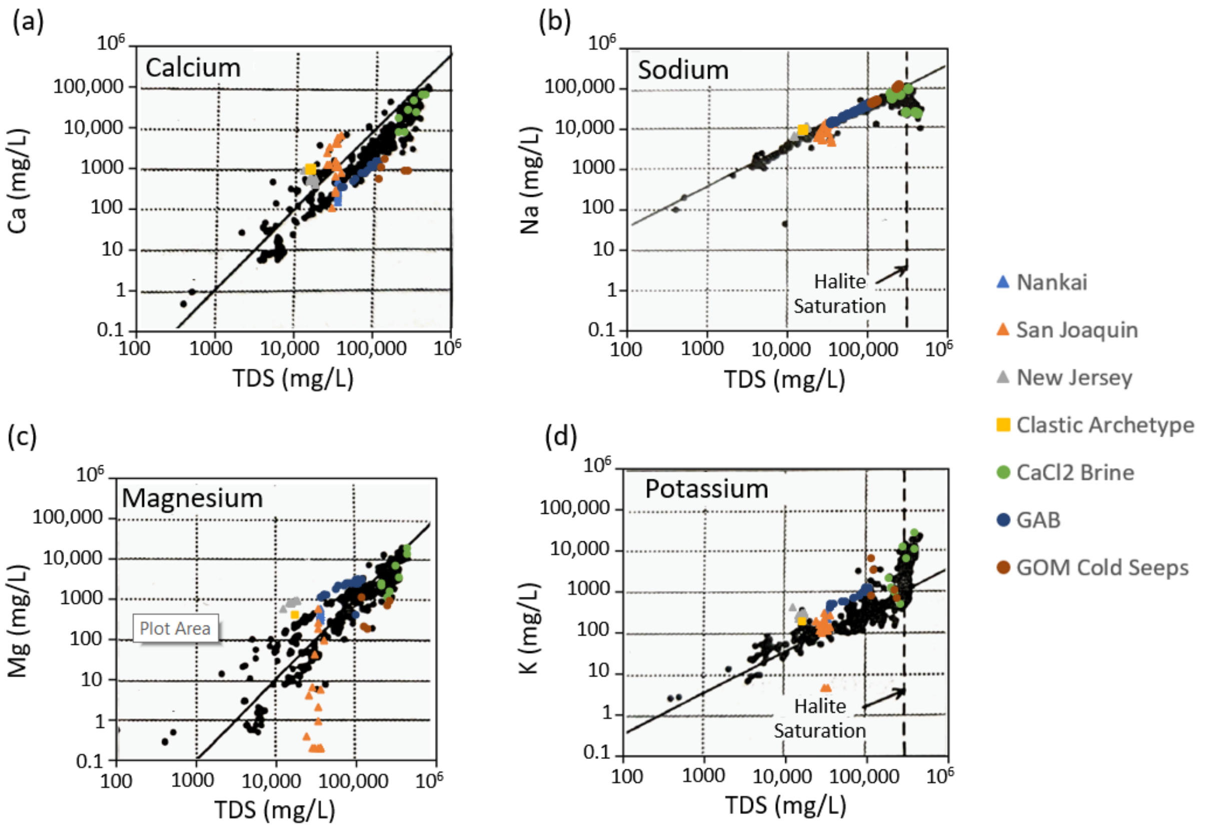

Supplementary Figure 3. Groundwater compositions for groundwater archetypes and selected sedimentary basins plotted over compilation of saline fluid compositions from sedimentary basins (black circles) by Hanor (1994)[1]. (a) Calcium, (b) Sodium, (c) Magnesium, (d) Potassium. Samples from the Nankai Basin[2] are from an accretionary wedge (IODP Leg 131) and are shown only for comparison with other clastic samples. San Joaquin samples are from Fisher and Boles[3]. New Jersey samples are from IODP Leg 313[4]. $CaCl_2$ brine indicates the samples used for the $CaCl_2$ brine archetype. GAB indicates samples used for the carbonate archetype, from the Great Australian Bight[5]. GOM Cold Seeps indicates samples used for the Cold Seep Archetype (see Table 4). TDS was estimated from chlorinity for samples where salinity or TDS measurements were missing.

Supplementary Figure 4

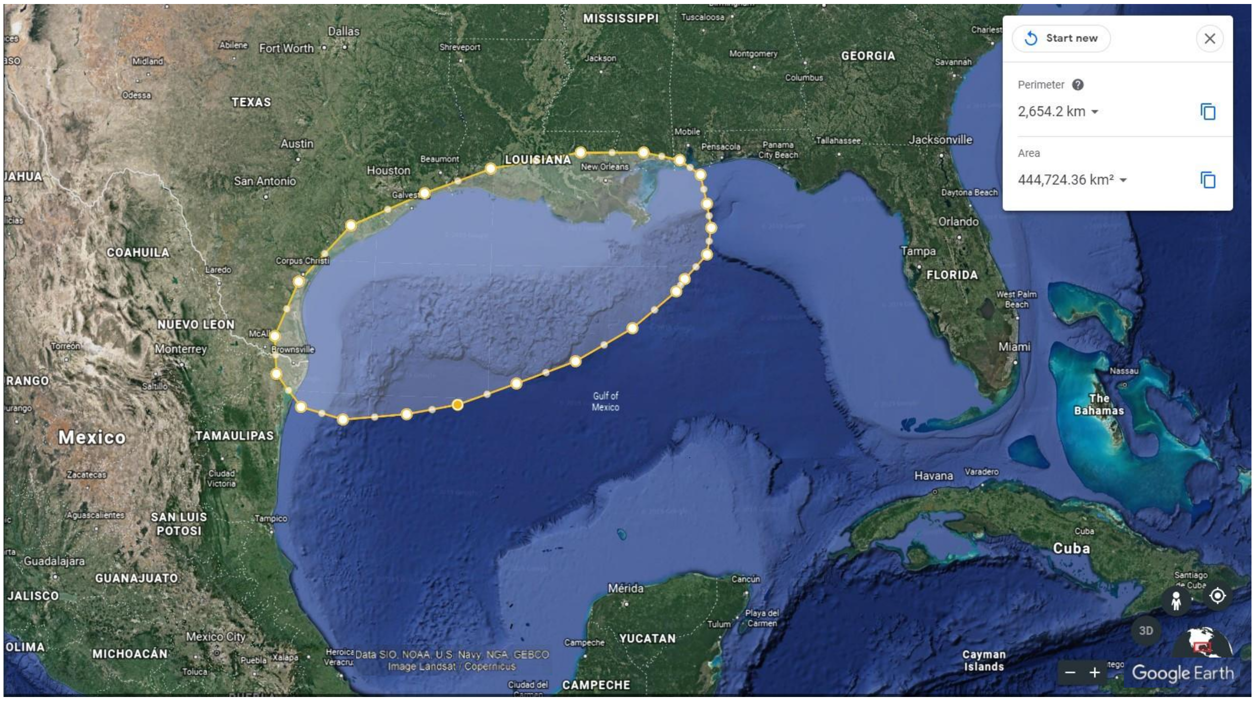

Supplementary Figure 4. Example area calculation for the Gulf of Mexico, representative of the area calculations for the basins representing compaction-driven flow. These areas were used to confirm areas reported in the references listed in Table 2. Figure created in Google Earth. Map image from Landsat/Copernicus. Map data from SIO, NOAA, U.S. Navy, NGA, GEBCO.

Supplementary References

1. Hanor, J. S. Origin of saline fluids in sedimentary basins. in *Geofluids: Origin, Migration and Evolution of Fluids in Sedimentary Basins* (ed. Parnell, J.) 151–174 (Geological Society Special Publication No. 78, 1994).
2. Geiskes, J. M., Gamo, T. & Kaster, M. Major and minor element geochemistry of interstitial waters of Site 808, Nankai Trough: An overview. in *Proceedings of the Ocean Drilling Program, Scientific Reports* vol. 131 387–394 (1993).
3. Fisher, J. B. & Boles, J. R. Water—rock interaction in Tertiary sandstones, San Joaquin basin, California, U.S.A.: Diagenetic controls on water composition. *Chem Geol* **82**, 83–101 (1990).
4. Mountain, G., Proust, J., McInroy, D., Cotterill, C. & Shipboard Scientific Party. Site M0027. in *Proceedings of the IODP* vol. 313 (Integrated Ocean Drilling Program Management Int., 2010).
5. Feary, D. A., Hine, A. C., Malone, M. J. & Shipboard Scientific Party. Site 1126. in *Proceedings of the ODP, Initial Reports* vol. 182 1–110 (2000).
